# Introdução da alimentação complementar e fatores associados em recém-nascidos pré-termo e com baixo peso: estudo de coorte prospectivo

**DOI:** 10.1590/0102-311XPT194923

**Published:** 2024-09-09

**Authors:** Priscilla Larissa Silva Pires, Rejane Sousa Romão, Rayany Cristina de Souza, Leandro Alves Pereira, Ana Elisa Madalena Rinaldi, Vivian Mara Gonçalves de Oliveira Azevedo

**Affiliations:** 1 Universidade Federal de Uberlândia, Uberlândia, Brasil.

**Keywords:** Recém-nascido Prematuro, Recém-nascido de Baixo Peso, Alimentação Complementar, Premature Infant, Low Birth Weight Infant, Complementary Feeding, Recién-nacido Prematuro, Recién-nacido de Bajo Peso, Alimentación Complementaria

## Abstract

O objetivo deste artigo foi analisar a associação entre os fatores sociodemográficos, as características maternas e neonatais e o tempo de introdução da alimentação complementar em recém-nascidos pré-termo e com baixo peso. Trata-se de um estudo de coorte prospectivo feito com 79 recém-nascidos pré-termo com peso menor ou igual a 1.800g. Os dados foram coletados no momento da alta hospitalar e ao 6º, 9º e 12º mês de idade gestacional corrigida (IGC), com auxílio de um questionário estruturado para analisar o tempo de introdução da alimentação complementar e texturas dos alimentos introduzidos. Além disso, para avaliar o risco de atraso de desenvolvimento, utilizou-se o *Survey of Well-being of Young Children* (SWYC-BR). Para análise das variáveis, aplicou-se regressão de riscos proporcionais de Cox. A introdução da alimentação complementar foi observada nos recém-nascidos pré-termo, com a mediana de idade de introdução de alimentos líquidos (3,50; IQ: 2,50-5,00), seguido por sólidos (4,70; IQ: 3,20-5,20) e pastosos (5,00; IQ: 4,50-5.50). Ainda, verificou-se associação da idade gestacional (RR = 1.25; IC95%: 1,02-1,52) em todo o processo da introdução alimentar. Para os alimentos sólidos e pastosos, aqueles com o maior tempo de internação (RR = 1,03; IC95%: 1,10- 1,05) e em amamentação mista (RR = 2,97; IC95%: 1,24-7,09) adiaram mais o tempo para introduzir a alimentação complementar. Para alimentos líquidos, recém-nascidos pré-termo menos graves (*Score for Neonatal Acute Physiology and Perinatal Extension* - SNAPPE II [RR = 0,96; IC95%: 0,94-0,98]) e mães que estavam amamentando na alta hospitalar (RR = 11,49; IC95%: 1,57-84,10) postergaram a introdução alimentar. Diretrizes para melhor orientação de profissionais e pais e/ou responsáveis sobre o momento ideal de introdução alimentar se faz necessário.

## Abstract (en)

This study aimed to analyze the association between sociodemographic factors, maternal and neonatal characteristics and the time taken to introduce complementary feeding in low birthweight and preterm newborns. This is a prospective cohort study of 79 preterm newborns weighing less than or equal to 1,800g. Data were collected at the time of hospital discharge and at the 6^th^, 9^th^ ,and 12^th^ months of corrected gestational age (CGA), using a structured questionnaire to analyze the time taken to introduce complementary feeding and the texture of the foods introduced. Furthermore, the *Survey of Well-being of Young Children* (SWYC-BR) was used to assess the risk of developmental delay. Cox proportional hazards regression was used to analyze the variables. The introduction of complementary feeding was assessed in preterm newborns based on the median age of introduction of liquid foods (3.50; IQR: 2.50-5.00), followed by solid (4.70; IQR: 3.20-5.20) and soft foods (5.00; IQR: 4.50-5.50). There was also an association with gestational age (RR = 1.25; 95%CI: 1.02-1.52) throughout the process of food introduction. For solid and soft foods, those with the longest length of stay (RR = 1.03; 95%CI: 1.10-1.05) and on mixed breastfeeding (RR = 2.97; 95%CI: 1.24-7.09) delayed the introduction of complementary feeding the longest. For liquid foods, less severe preterm newborns (*Score for Neonatal Acute Physiology and Perinatal Extension* - SNAPPE II [RR = 0.96; 95%CI: 0.94-0.98]) and mothers who were breastfeeding at hospital discharge (RR = 11.49; 95%CI: 1.57-84.10) delayed the introduction of complementary feeding. Guidelines are needed to better advise professionals and parents and/or guardians on the ideal time to introduce feeding.

## Abstract (es)

El objetivo de este estudio fue analizar la asociación entre los factores sociodemográficos, características maternas y neonatales y el momento de introducción de la alimentación complementaria en recién nacidos pretérmino (recém-nascidos pré-termo) y de bajo peso. Se trata de un estudio de cohorte prospectivo realizado con 79 recém-nascidos pré-termo con un peso menor o igual a 1.800g. Los datos se recopilaron en el momento del alta hospitalaria y al 6º, 9º y 12º mes de edad gestacional corregida (EGC), con la ayuda de un cuestionario estructurado para analizar el momento de introducción de la alimentación complementaria y las texturas de los alimentos introducidos. Además, para evaluar el riesgo de retraso en el desarrollo, se utilizó la *Survey of Well-being of Young Children* (SWYC-BR). Para analizar las variables, se aplicó la regresión de riesgos proporcionales de Cox. La introducción de la alimentación complementaria se observó en los recém-nascidos pré-termo, con la mediana de edad de introducción de alimentos líquidos (3,50; IIC: 2,50-5,00), seguido de los sólidos (4,70; IIC: 3,20-5,20) y pastosos (5,00; IIC: 4,50-5,50). Además, se constató la asociación de la edad gestacional (RR = 1,25; IC95%: 1,02-1,52) durante todo el proceso de introducción alimentaria. En el caso de alimentos sólidos y pastosos, aquellos con mayor tiempo de hospitalización (RR = 1,03; IC95%: 1,10-1,05) y en lactancia mixta (RR = 2,97; IC95%: 1,24-7,09) retrasaron más la introducción de alimentación complementaria. En el caso de alimentos líquidos, los recém-nascidos pré-termo menos graves (*Score for Neonatal Acute Physiology and Perinatal Extension* - SNAPPE II [RR = 0,96; IC95%: 0,94-0,98]) y las madres que estaban amamantando al alta hospitalaria (RR = 11,49; IC95%: 1,57-84,10) pospusieron la introducción de alimentos. Se hacen necesarias pautas para una mejor orientación a profesionales y padres o tutores sobre el momento ideal para la introducción alimentaria.

## Introdução

A prematuridade é considerada um problema de saúde pública por ser uma das principais causas de morbimortalidade infantil ^1^. Estima-se que a cada ano ocorram 15 milhões de nascimentos prematuros no mundo ^2^.

Sabe-se que os recém-nascidos pré-termo - idade gestacional < 37 semanas, e com baixo peso (< 2.500g) ^2^, apresentam maiores riscos em relação aos recém-nascidos a termo, de atraso no crescimento e desenvolvimento devido à imaturidade fisiológica ^3^ ^,^ ^4^. Além disso, são mais propensos a desenvolver distúrbios metabólicos e nutricionais ^5^. Sendo assim, a nutrição infantil nos primeiros mil dias é crucial para o crescimento e desenvolvimento global adequados, desde a infância até a vida adulta ^6^ ^,^ ^7^.

O leite materno é considerado a principal fonte de nutrição para os lactentes, uma vez que propicia todos os nutrientes essenciais para o crescimento e desenvolvimento saudável, fornece aporte imunológico que atua na prevenção de doenças como infecções respiratórias, enterocolite necrosante, anemia e diarreia, e ainda possibilita o fortalecimento do vínculo afetivo familiar ^8^ ^,^ ^9^. Em relação aos recém-nascidos pré-termo, esses benefícios possibilitam ainda redução no tempo de internação e reinternações, menores taxas de infecção, aumento do desempenho neuropsicomotor e melhor prognóstico clínico ^5^ ^,^ ^10^ ^,^ ^11^.

A Organização Mundial da Saúde (OMS) recomenda que o leite materno seja oferecido de forma exclusiva até os seis meses de vida e complementado até dois anos ou mais ^12^. Entretanto, orientações sobre o momento ideal de início da introdução da alimentação complementar em recém-nascidos pré-termo ainda são escassas na literatura ^13^.

Além disso, o aleitamento materno é considerado um aliado na introdução da alimentação complementar. Estudos mostram que o leite materno proporciona a experiência de sabores e aromas dos alimentos consumidos pela nutriz para o lactente. Assim, as crianças amamentadas apresentam melhor adesão à introdução da alimentação complementar ^14^ ^,^ ^15^ ^,^ ^16^.

Os recém-nascidos pré-termo apresentam maiores dificuldades alimentares devido aos fatores relacionados à imaturidade fisiológica, como instabilidade cardiorrespiratória, distúrbios metabólicos, sucção, deglutição e respiração descoordenados e diminuição do tônus oromotor ^5^. Pesquisas indicam que, ao iniciar a introdução da alimentação complementar, os recém-nascidos pré-termo podem apresentar comportamentos defensivos durante a oferta de alimentos, como seletividade e recusa alimentar ^17^ ^,^ ^18^. Entretanto, ainda há escassez de estudos que avaliem o momento ideal para a introdução da alimentação complementar nessa população, bem como os fatores que podem influenciar na adaptação nutricional dos recém-nascidos pré-termo. Tais estudos podem auxiliar tanto os profissionais de saúde quanto os pais nesse período de transição.

Dessa forma, o objetivo deste texto foi analisar a associação entre os fatores sociodemográficos, as características maternas e neonatais e o tempo de introdução da alimentação complementar em recém-nascidos pré-termo e com baixo peso.

## Método

### Desenho do estudo e amostra

Trata-se de um estudo de coorte prospectivo feito com recém-nascidos pré-termo que foram internados na unidade neonatal do Hospital de Clínicas da Universidade Federal de Uberlândia (HC-UFU). O HC-UFU atua como principal porta de acesso à saúde pública da região, principalmente para o atendimento de urgência e emergência e de alta complexidade, fornecendo serviço de saúde vinculado apenas ao Sistema Único de Saúde (SUS). O período de coleta de dados ocorreu entre maio de 2021 e setembro de 2022.

Para a composição da amostra de pesquisa, foram incluídos os recém-nascidos pré-termo com peso de nascimento igual ou inferior a 1.800g, nascidos no hospital onde foram feitas as coletas de dados, que estiveram internados na unidade neonatal e cujas mães aceitaram participar da pesquisa. Este estudo foi iniciado juntamente com outro estudo multicêntrico de amostra semelhante, sendo esse o motivo pela escolha dos recém-nascidos pré-termo especificamente com peso igual ou abaixo de 1.800g.

Além disso, considerou-se como critérios de elegibilidade: ausência de síndromes genéticas, asfixia perinatal, gemelaridade, malformação congênita grave, doença metabólica e mães que apresentassem doenças psiquiátricas, toxicodependência ou qualquer outra condição que impossibilitasse a amamentação, como a exposição materna ao HIV. Entre os critérios de exclusão, foram considerados os óbitos neonatais e maternos, recusa materna, além dos recém-nascidos pré-termo que tiveram diagnóstico de alteração do sistema nervoso central durante o período de coleta de dados.

**Figura 1:**
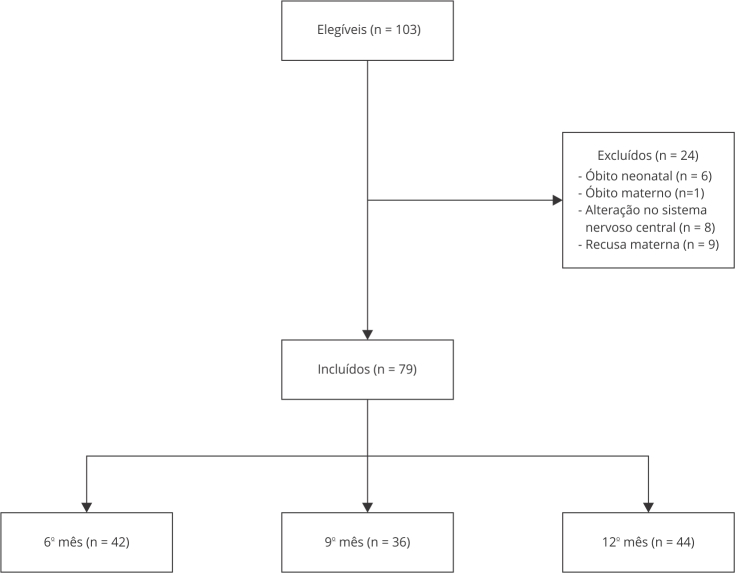
Fluxograma de seleção dos participantes do estudo.

Dos 103 recém-nascidos pré-termo elegíveis para o estudo, 79 foram incluídos na amostra final (Figura 1). Para validação do tamanho da amostra atingida, o cálculo amostral foi feito considerando 95% de confiança e cálculo conservativo (ausência de prevalência histórica). Foi atingida margem de erro de 7,9%, considerada suficiente e, portanto, o tamanho amostral de 79 recém-nascidos pré-termo foi mantido.

### Coleta de dados

Inicialmente, as mães foram abordadas na maternidade do hospital até 24 horas após o parto, convidadas a participar da pesquisa, e aquelas que consentiram assinaram o Termo de Consentimento Livre Esclarecido (TCLE).

Os questionários do estudo foram enviados nos seguintes períodos: no momento da alta hospitalar, ao 6º, 9º e 12º mês de idade gestacional corrigida (IGC). Utilizou-se o Google Forms (https://workspace.google.com/) por meio eletrônico (via WhatsApp e/ou *e-mail*) para a coleta de dados das mães que tinham acesso à internet, e ligações telefônicas para aquelas que não tinham acesso. Ainda, foram coletadas informações maternas e neonatais obtidas por meio do prontuário eletrônico e físico solicitados ao setor de arquivo institucional.

No momento da alta hospitalar, as mães responderam ao formulário online sobre os dados sociodemográficos (idade, escolaridade), obstétricos (tipo de parto, paridade) e econômicos (trabalho materno, licença maternidade). No sexto, nono e décimo segundo meses de IGC, elas responderam às questões referentes à amamentação (se estavam amamentando, se faziam uso de mamadeira, copinho e/ou translactação), licença maternidade (sim/não), momento da introdução da alimentação complementar (*checklist* alimentar) e desenvolvimento neuropsicomotor dos recém-nascidos pré-termo (escala *Survey of Well-being of Young Children* - SWYC-BR) ^19^.

### Variáveis de desfechos

O principal desfecho do estudo foi o tempo de introdução da alimentação complementar dos recém-nascidos pré-termo. Para isso, aplicou-se um questionário semiestruturado composto por um *checklist* alimentar que continha perguntas sobre o primeiro mês de oferta dos alimentos líquidos, sólidos e pastosos - “indique a idade do seu bebê quando foi ofertado pela primeira vez cada um dos alimentos citados abaixo”.

O *checklist* alimentar foi composto pelos seguintes itens: (1) líquidos - água, água com açúcar, chá, outros leites (caixinha, saquinho), fórmula infantil, suco de frutas, suco em pó/caixinha, refrigerante, café; (2) pastosos - papinhas caseiras, papinhas industrializadas, mingau, mingau com leite; e (3) sólidos - frutas, frutas alaranjadas, bala e pirulito, bolacha e salgadinho de pacote, macarrão instantâneo, carne, fígado, ovo, feijão, arroz e tubérculos (batata, inhame, mandioca, cará), legumes, legumes de cor alaranjada, verduras, salsicha, linguiça, *nuggets*.

Optamos por adotar como premissa que alimentos amassados tinham consistência de pastosos, uma vez que os termos sólido, semissólido e pastoso não são de fácil compreensão para as mães ^20^. Além disso, a fim de melhorar a acurácia do relato da consistência dos alimentos, foram anexadas fotos nos questionários enviados mostrando exemplos de alimentos sólidos, líquidos e pastosos. Ressaltamos que todas as informações coletadas foram baseadas nos relatos e percepções dos pais e/ou responsáveis.

### Variáveis de exposição

Consideramos as características neonatais e maternas, bem como o desenvolvimento neuropsicomotor.

Os dados neonatais investigados foram o sexo, a idade gestacional (semanas), o peso ao nascimento (gramas) e o tipo de alimentação ofertada na alta hospitalar (aleitamento materno exclusivo, aleitamento materno misto ou fórmula infantil). Também foram analisados o tempo de internação (dias) e o *Score for Neonatal Acute Physiology and Perinatal Extension* (SNAPPE-II), obtidos por meio do prontuário eletrônico e físico solicitado ao setor de arquivo institucional ^21^. 

O SNAPPE-II é uma escala desenvolvida para mensurar a gravidade e o risco de mortalidade em recém-nascidos que necessitam de cuidados na unidade de terapia intensiva neonatal (UTI neonatal). Esta escala é constituída por nove itens: pressão arterial média, temperatura na admissão na UTI neonatal, índice de oxigenação (PaO_2_/FiO_2_), pH sanguíneo, presença de convulsões múltiplas, volume urinário, peso de nascimento, adequação para a idade gestacional e escore de APGAR no 5º minuto de vida - sendo que quanto maior a pontuação obtida, maior a gravidade do recém-nascidos ^21^.

Sabendo que o desenvolvimento neuropsicomotor do recém-nascidos pré-termo influencia na transição alimentar segura, este também foi avaliado utilizando o SWYC-BR ^19^, um instrumento de triagem para alterações do desenvolvimento e do comportamento infantil, validado para a população brasileira. Trata-se de um questionário com perguntas direcionadas aos pais/cuidadores, que avalia os marcos do desenvolvimento cognitivo, linguagem e motor, bem como os sintomas comportamentais e emocionais, além da insegurança alimentar no ambiente familiar da criança. O questionário também investiga as percepções e preocupações dos pais quanto ao comportamento, aprendizado e desenvolvimento do filho. Os riscos no desenvolvimento neuropsicomotor dos recém-nascidos pré-termo foram avaliados em três momentos (6º, 9º e 12º mês de IGC).

### Análise de dados

A normalidade dos dados numéricos foi analisada pelo teste de Shapiro-Wilk. A análise descritiva das variáveis demográficas, socioeconômicas, relativas ao parto, à saúde materna e infantil e à introdução alimentar foi desenvolvida para a amostra completa. Os dados qualitativos foram expressos em frequências absoluta e relativa, enquanto os dados quantitativos foram apresentados em média e desvio padrão ou mediana e intervalo interquartil (IQ).

Para analisar a associação entre as variáveis sociodemográficas, as características maternas e neonatais e a idade mediana dos alimentos introduzidos, foi realizada a regressão de riscos proporcionais de Cox. As análises estatísticas foram feitas no software Stata SE, versão 14 (https://www.stata.com). O nível de significância adotado foi de 5% em todos os testes.

Por se tratar de uma pesquisa longitudinal, houve perdas durante o seguimento (Figura 1). No entanto, ao analisar as principais características da amostra perdida com os que permaneceram no estudo (peso ao nascimento, sexo, idade gestacional, SNAPPE-II, idade materna, escolaridade materna, dieta na alta hospitalar e tempo de internação), não foi observada diferença estatisticamente significante (p > 0,05).

Além disso, para minimizar o viés de informação, uma vez que se trata de uma pesquisa com dados autorrelatados, foi elaborada uma análise de consistência para os valores relatados de idade de introdução de alimentos ao longo do tempo (três momentos diferentes), por meio do coeficiente de correlação intraclasse (CCI), obtendo-se concordância considerada ótima (valores entre 0,75 e 1,0).

### Aspectos éticos

A pesquisa foi submetida ao Comitê de Ética em Pesquisa com Seres Humanos da Universidade Federal de Uberlândia (parecer nº 4.312.356).

## Resultados

Da amostra de 79 recém-nascidos pré-termo, 58% (n = 46) eram do sexo feminino com média de peso ao nascimento de 1.249,2 ± 346,8g. A mediana de tempo de internação hospitalar foi de 48 dias (IQ: 31-65 dias). A pontuação média do SNAPPE-II foi de 17,7 ± 16,7 pontos. No momento da alta, 64% dos recém-nascidos pré-termo estavam em aleitamento materno misto (Tabela 1); destes, 25% iniciaram a introdução da alimentação complementar antes dos quatro meses de IGC.

Quanto aos dados maternos, a média da idade materna foi de 26,6 ± 6,5 anos e 43% (n = 34) cursaram até o Ensino Médio. O tipo de parto predominante foi o cesáreo (73%; n = 58). As demais características dos recém-nascidos pré-termo e suas mães estão apresentadas na Tabela 1.

**Tabela 1:** Caracterização sociodemográfica de recém-nascidos pré-termo e suas mães (N = 79).

Variáveis	Média ± DP	Mediana (IQ)	Teste de Shapiro-Wilk (valor de p)
Peso ao nascer (gramas)		1230 (1.007,5-1.577,5)	0,12 (< 0,01)
Idade gestacional (semanas)	30,4 ± 2,8	30,5	0,10 (0,27)
SNAPPE-II		13 (5-25)	0,16 (< 0,01)
Tempo de internação (dias)	-	48 (31-65)	0,12 (< 0,01)
Características neonatais	n	%	
Sexo			
Feminino	46	58	
Masculino	33	41	
Tipo de dieta na alta			
Aleitamento materno + fórmula complementar	51	64	
Aleitamento materno exclusivo	26	32	
Somente fórmula	2	2	
Participação na Unidade de Cuidado Intermediário Neonatal Canguru	56	69	
Características maternas	n	%	
Escolaridade			
Ensino Médio	34	43	
Ensino Superior	33	41	
Ensino Fundamental	8	10	
Não informado	4	5	
Tipo de parto			
Cesárea	58	73	
Vaginal	21	26	
Mês de coleta de dados	6º (%)	9º (%)	12º (%)
Mães trabalhando (sim)	31	25	31
Mães amamentando (sim)	39	33	21

DP: desvio padrão; IQ: intervalo interquartil; SNAPPE-II: *Score for Neonatal Acute Physiology and Perinatal Extension*.

Ao analisar o início da introdução da alimentação complementar, observou-se menor mediana para a oferta de alimentos líquidos, sendo que até 3,5 meses, 50% dos recém-nascidos pré-termo tinham consumido esses alimentos (mediana = 3,50; IQ: 2,50-5,00 meses). Já para os alimentos sólidos, 50% das crianças tinham recebido esses alimentos até 4,7 meses (mediana = 4,70; IQ: 3,20-5,20 meses) e pastosos até 5 meses (mediana = 5,00; IQ: 4,50-5,50 meses) (Tabela 2). Com relação ao aleitamento materno exclusivo, a duração mediana foi semelhante ao de outros alimentos líquidos (fórmula infantil, outros leites e água), sendo que até 3,5 meses, 50% dos recém-nascidos de baixo peso estavam sendo amamentados exclusivamente.

**Tabela 2:** Tempo de introdução (meses) da alimentação complementar nos recém-nascidos pré-termo avaliados (N = 79).

Variáveis	Mediana (IQ)
Líquidos	3,50 (2,50-5,00)
Sólidos	4,70 (3,20-5,20)
Pastosos	5,00 (4,50-5,50)

IQ: intervalo interquartil.

Na Tabela 3 são apresentados os resultados referentes às análises de associação entre as características neonatais e maternas e o tempo para a introdução da alimentação complementar. Houve associação entre a idade gestacional e a oferta de todos os tipos de alimento, sendo que a idade de introdução dos alimentos foi maior para recém-nascidos pré-termo com maior idade gestacional. Para os alimentos pastosos e sólidos, houve associação em relação ao tempo de internação (razão de risco - RR = 1,03; intervalo de 95% de confiança - IC95%: 1,10-1,05), em que quanto maior o tempo de internação, maior a idade de introdução da alimentação complementar. Recém-nascidos pré-termo que estavam sendo amamentados na alta hospitalar apresentaram maior mediana de introdução dos alimentos líquidos. Entretanto, ao avaliar os recém-nascidos pré-termo menos graves (SNAPPE-II), observou-se associação negativa (RR = 0,96; IC95%: 0,94-0,98), em que quanto menor o valor de SNAPPE-II, ou seja, menor gravidade clínica, maior o tempo para introduzir os alimentos líquidos. Além disso, mães que mantiveram a amamentação após a alta hospitalar (RR = 2,22; IC95%: 1,03-4,75) também adiaram a introdução da alimentação complementar (alimentos líquidos e pastosos).

**Tabela 3:** Associação entre as características neonatais e maternas e o tempo de introdução alimentar complementar (N = 79).

Consistência dos alimentos	RR (IC95%)	Valor de p
Sólido		
Idade gestacional (semanas)	1,26 (1,05-1,52)	0,01 *
Tempo de internação (dias)	1,03 (1,00-1,05)	< 0,01 *
Líquido		
Idade gestacional (semanas)	1,25 (1,02-1,52)	0,06
Tipo de dieta na alta (**leite materno** + fórmula)	11,49 (1,57-84,01)	0,04 *
Amamentação (**sim**)	2,22 (1,03-4,75)	0,08
SNAPPE-II (escore)	0,96 (0,94-0,98)	< 0,01 *
Pastoso		
Idade gestacional (semanas)	1,29 (1,07-1,55)	0,02 *
Tempo de internação (dias)	1,03 (1,00-1,05)	0,01 *
Está amamentando (**sim**)	2,97 (1,24-7,09)	0,03 *

IC95%: intervalo de 95% de confiança; RR: razão de risco; SNAPPE-II: *Score for Neonatal Acute Physiology and Perinatal Extension*.

Nota: as categorias em negrito foram consideradas como referência. Teste de regressão de riscos proporcionais de Cox.

* Valores significativos (p < 0,05).

Em relação à avaliação do desenvolvimento infantil, observou-se predominância de risco de atraso comportamental (64,1%; n = 25), seguido pelo risco de atraso no desenvolvimento (53,8%; n = 21), insegurança alimentar (10,2%; n = 4), e riscos psicossociais (17,9%; n = 7), no 6º mês de IGC. No 9º mês, houve uma semelhança entre o risco de atraso comportamental e de desenvolvimento (44,8%; n = 14), seguido pelo risco psicossocial (24,1%; n = 7) e uma queda no índice de insegurança alimentar (6,9%; n = 2). Já no 12º mês, observou-se um percentual semelhante de risco de atraso comportamental e risco psicossocial (35,9%; n = 16), com uma queda no risco de atraso no desenvolvimento (12,8%; n = 9) e na insegurança alimentar (10,2%; n = 8). No entanto, não foi observada associação entre o risco de atraso tanto no comportamento quanto no desenvolvimento e o tempo de introdução de alimentação complementar (p > 0,05) (dados não apresentados em tabelas).

## Discussão

Observou-se, neste estudo, que em relação aos fatores preditores da introdução alimentar complementar dos recém-nascidos pré-termo, houve associação entre idade gestacional e o momento de início da oferta de todos os tipos de alimento, sendo que a idade de introdução dos alimentos foi maior para recém-nascidos pré-termo com maior idade gestacional. Na introdução dos alimentos sólidos e pastosos, os lactentes com maior tempo de internação demoraram mais para introduzir a alimentação complementar. Quanto aos alimentos líquidos, os recém-nascidos pré-termo menos graves e as mães que estavam amamentando na alta hospitalar postergaram a introdução da alimentação complementar. Além disso, as mães que optaram por manter a amamentação, adiaram a introdução da alimentação complementar (alimentos líquidos e pastosos).

A introdução da alimentação complementar nos recém-nascidos pré-termo analisados se iniciou com oferta de líquidos a partir do 3º mês de IGC e de sólidos e pastosos a partir do 4º mês de IGC. Usualmente, os recém-nascidos pré-termo iniciam a alimentação complementar, especialmente com os sólidos, antes dos recém-nascidos a termo ^5^ ^,^ ^22^ ^,^ ^23^ ^,^ ^24^ ^,^ ^25^ ^,^ ^26^ ^,^ ^27^, sobretudo por influências de fatores maternos como idade e baixa escolaridade, além dos fatores socioculturais ^22^ ^,^ ^23^. Em relação às influências culturais, citamos a sensação de leite materno insuficiente, crenças sobre a complementação do leite materno com o uso de fórmula para melhorar a nutrição ^25^, comportamento do recém-nascidos pré-termo (interpretação de que o choro significa fome) ^24^ e pais que consideram a idade cronológica para iniciar a alimentação complementar ^22^. Embora haja recomendações, como as da Sociedade Brasileira de Pediatria (SBP) ^17^, Sociedade Europeia de Gastroenterologia Pediátrica, Hepatologia e Nutrição (ESPGHAN) ^28^ e Associação Americana de Pediatria ^29^, sobre o início da alimentação complementar entre quatro a seis meses de IGC para os recém-nascidos pré-termo, as recomendações da OMS estabelecem seis meses, porém com foco nos recém-nascidos a termo, sem consenso sobre o início para os recém-nascidos pré-termo ^16^.

Estudos prévios orientam iniciar a introdução de alimentos sólidos a partir dos três meses de IGC, considerando a aquisição de habilidades motoras, pois estas permitem o consumo desses alimentos ^5^ ^,^ ^22^ ^,^ ^24^ ^,^ ^25^. A ESPGHAN ^28^ recomenda a introdução de alimentos sólidos a partir do quarto mês de IGC, e a Associação Americana de Pediatria ^29^ sugere o início da alimentação complementar entre cinco e oito meses de idade cronológica, considerando o desenvolvimento motor, aquisição do paladar e prontidão para introdução de texturas alimentares. A SBP orienta que, para aqueles recém-nascidos pré-termo com uso exclusivo de fórmula infantil, a alimentação complementar pode ser iniciada aos três meses de IGC ^17^, já o *Guia Alimentar para Crianças Brasileiras Menores de 2 Anos* ^30^, com recomendações especialmente para recém-nascidos a termo, orienta a introdução dos alimentos a partir dos seis meses, mesmo em uso de fórmula. Em nosso estudo, dentre os 51 recém-nascidos pré-termo que estavam em uso de fórmula, 20 iniciaram a introdução alimentar antes dos quatro meses de IGC.

Estudos prévios constataram benefícios em iniciar a introdução da alimentação complementar entre sete a dez meses de IGC para os recém-nascidos pré-termo, como menor recorrência de internação hospitalar ^22^ ^,^ ^31^. No entanto, assinalam os riscos de alteração no comportamento durante a alimentação complementar, com possível recusa alimentar ^5^ ^,^ ^22^. Em contrapartida, há evidências de risco de sobrepeso e obesidade infantil para aqueles que iniciam antes dos seis meses de IGC ^32^ ^,^ ^33^.

Observamos também que mães que mantiveram o aleitamento materno postergaram a introdução da alimentação complementar. Sabe-se que o aleitamento materno é apontado como aliado na introdução da alimentação complementar, principalmente por fornecer aromas e sabores adquiridos pela nutriz ao lactente, auxiliando em melhor adesão na fase da oferta de alimentos ^16^ ^,^ ^34^. Entretanto, a introdução precoce da alimentação complementar pode ocasionar o desmame, o que pode oferecer riscos ao recém-nascido, como obesidade tanto na infância quanto na fase adulta; maior propensão a alergias, doenças gastrointestinais e diarreia, infecções respiratórias e dermatológicas; ainda interfere na absorção de nutrientes, o que causa deficiências nutricionais e impacto negativo na saúde infantil ^10^ ^,^ ^27^ ^,^ ^35^. Estudo prévio mostrou que mães que amamentam tendem a evitar o uso de fórmula e optam por adiar a introdução da alimentação complementar. No entanto, os autores sinalizam quanto aos riscos de déficit nutricional quando há demora na oferta de outros alimentos ^25^. Ressalta-se a importância de incentivar a amamentação, principalmente dentro das UTIN, a fim de proporcionar ao recém-nascidos pré-termo os benefícios do aleitamento materno, o fortalecimento do vínculo mãe-bebê e a permanência da amamentação pós-alta.

Outro resultado observado foi a associação entre o tempo de internação e a introdução da alimentação complementar, em que quanto maior o tempo de internação, mais tempo os recém-nascidos pré-termo demoraram para iniciar outros tipos de alimentos, especificamente os sólidos e pastosos. Há relatos de que recém-nascidos pré-termo que recebem leite materno ficam menos tempo em internação hospitalar em relação àqueles alimentados por fórmula ^36^. Além disso, acredita-se que a internação prolongada ocorreu devido à gravidade clínica do recém-nascidos pré-termo, o que pode prorrogar o início da introdução de alimentos sólidos e pastosos.

Os recém-nascidos pré-termo menos graves (escore de SNAPPE-II mais baixo) demoraram para introduzir alimentos líquidos. Possivelmente, os recém-nascidos pré-termo mais estáveis permaneceram mais tempo em amamentação e, por isso, demoraram mais tempo para introduzir a alimentação complementar. Além disso, a exposição do recém-nascidos pré-termo a procedimentos invasivos e a maior permanência em UTIN podem ocasionar alterações no seu desenvolvimento motor e comportamentos de recusa durante a introdução da alimentação complementar ^5^ ^,^ ^21^. Ademais, recém-nascidos pré-termo mais graves são mais propensos a receberem fórmulas infantis durante a hospitalização ^18^, o que seria um preditor para o início precoce da introdução da alimentação complementar ^25^.

Ainda, não observamos associação entre as variáveis idade e escolaridade materna e alteração no comportamento e desenvolvimento dos recém-nascidos pré-termo com a introdução da alimentação complementar. Em contrapartida, estudos publicados evidenciaram que menor a idade materna e baixo grau de escolaridade influenciaram no desmame do aleitamento materno e início da introdução da alimentação antes dos seis meses de vida ^22^ ^,^ ^23^.

A avaliação do desenvolvimento dos recém-nascidos pré-termo para o início da introdução da alimentação complementar é de extrema importância para garantir uma transição segura. Em nosso estudo, verificou-se predominância do risco de atraso no comportamento e desenvolvimento dos recém-nascidos pré-termo. Recomendações e diretrizes publicadas orientam considerar fatores como desenvolvimento motor oral e do paladar, além de prontidão para explorar novas texturas ^6^ ^,^ ^22^. Além das alterações motoras, episódios frequentes de choro, irritação, extensão corporal, náuseas, engasgos, tosses, vômitos e recusa alimentar apresentam impacto negativo na alimentação, apetite e crescimento dessas crianças ^17^.

As principais limitações identificadas foram a realização da coleta de dados durante o período pandêmico, o que fez necessário a adequação das entrevistas por meio de formulários *online*. Além disso, o momento da introdução da alimentação complementar foi baseado no relato materno. No entanto, para minimizar o risco de viés de memória, aplicou-se o questionário em três momentos distintos e foram anexadas fotos nos questionários enviados, exemplificando alimentos com aspecto sólido, líquido e pastoso. 

## Conclusão

Os resultados deste artigo mostraram que quanto maior a idade gestacional maior o tempo para início da alimentação complementar. Na introdução dos alimentos sólidos e pastosos, aqueles recém-nascidos pré-termo com maior tempo de internação demoraram mais tempo para introduzir a alimentação complementar. Além disso, as mães que optaram por manter a amamentação, adiaram a introdução da alimentação complementar (alimentos líquidos e pastosos).

Novos estudos ainda são necessários para que tenhamos respaldo científico no intuito de elaborarmos diretrizes na orientação à profissionais e pais e/ou responsáveis sobre o momento ideal da introdução da alimentação complementar, especialmente para recém-nascidos pré-termo.
